# Ayurvedic Treatment for Lumbar Disc Herniation With Radiculopathy: Protocol for a Randomized Controlled Trial

**DOI:** 10.2196/82936

**Published:** 2026-09-18

**Authors:** Aswani P S, Amit Kumar Rai, Bhagwan Sahai Sharma, Azeem Ahmad, Pratap Shankar Kizhakke Meladam, Rohit K S, Lisha S Raj, Rakesh Rana, Bhogavalli Chandra Sekhara Rao, Narayanam Srikanth, Rabinarayan Acharya

**Affiliations:** 1 National Ayurveda Research Institute for Panchakarma, under Central Council for Research in Ayurvedic Sciences (CCRAS), Ministry of Ayush, Government of India Thrissur, Kerala India; 2 CCRAS-Regional Ayurveda Research Institute Thiruvananthapuram, Kerala India; 3 A & U Tibbia College and Hospital New Delhi India; 4 Central Council for Research in Ayurvedic Science Janakpuri, Delhi India

**Keywords:** Ayurvedic treatment protocol, lumbar disc herniation, radiculopathy, pelvic traction, conservative management

## Abstract

**Background:**

The leading cause of disability worldwide is low back pain. The incidence of herniated disc is approximately 5 to 20 cases per 1000 adults per year, and herniated disc is prevalent in people in their third to fifth decade of life, with a male-to-female ratio of 2:1. In Ayurveda, the condition *grudhrasi* is considered analogous to radiculopathy. In conventional medicine, the first line of treatment includes conservative management, such as patient education, manual therapy, and nonsteroidal anti-inflammatory drugs. Ayurvedic treatments are used to manage lumbar radiculopathy.

**Objective:**

This clinical study aims to compare the safety and efficacy of an Ayurvedic treatment protocol with standard care in patients with lumbar disc herniation with radiculopathy.

**Methods:**

This is an open-label, randomized, controlled, parallel group clinical trial with a sample size of 80 participants who will be randomly allocated to 2 groups. The intervention group will receive an Ayurvedic treatment protocol, and the control group will be treated with conventional care, including physiotherapy, for a period of 60 days. The changes in the numeric pain rating scale and visual analog scale score and the Oswestry Disability Index (version 2.0) score will be considered the primary outcome measures and will be recorded at baseline (day 1) and on day 8 (after completing inpatient [IP] treatment), day 30, and day 60. The secondary outcome, quality of life, will be recorded at baseline (day 1) and on day 8 (after completing IP treatment), day 30, and day 60. The occurrence of adverse events and the need for rescue analgesic medications will be considered other secondary outcome measures and will be recorded on day 8 (after completing IP treatment), day 30, and day 60.

**Results:**

The study was funded in March 2023, and the study period is 36 months. Participant enrollment began in November 2023. A total of 136 participants were screened, of whom 80 were enrolled in the study. Data analysis is in progress, and the study outcomes are expected to be published by December 2026.

**Conclusions:**

The outcome of this trial may provide scientific evidence regarding the safety and efficacy of Ayurvedic treatment for lumbar disc herniation with radiculopathy. The study may also contribute to the development of evidence-based integrative management strategies and standardized treatment protocols for this condition.

**Trial Registration:**

Clinical Trial Registry of India CTRI/2023/05/052859; https://tinyurl.com/y6h4b5kb

**International Registered Report Identifier (IRRID):**

DERR1-10.2196/82936

## Introduction

The single leading cause of disability worldwide is low back pain; 60% to 80% of adults experience low back pain in varying degrees. Although the etiology and pathology are complex, they are strongly associated with intervertebral disc degeneration [1,2]. The incidence of herniated disc is approximately 5 to 20 cases per 1000 adults per year and is prevalent in people in their third to fifth decade of life, with a male-to-female ratio of 2:1 [3,4]. Approximately 40% of low back pain is of radicular origin and comes under the umbrella of sciatic syndrome [5]. Comprehensive Ayurveda textbooks mention a disease called *grudhrasi*, which is one of 80 types of disorders caused by vitiated *vata* (the regulatory functional factor of the body responsible for movement and cognition). The disease name refers to the characteristic gait of the patient, which resembles that of a vulture (*grudham*), with the affected leg becoming tense and curved. *Grudhrasi* is described as having 2 types: *vata dosha*–predominant and *vata-kapha–dosha* predominant. The main symptom of *grudhrasi* is pain that begins in the gluteal region (*sphik*) and radiates down the back, thigh, knee, calf, and foot, accompanied by stiffness, pricking pain, and twitching, resulting in restricted movement of the leg. In addition to these symptoms, aversion to food, drowsiness, and a feeling of heaviness may also occur [6-9].

As per Ayurveda, *vasti* or *basti* (medicated enema) is the prime treatment modality for disorders due to vitiated *vata dosha*. In this treatment, the ingredients are blended into an emulsion and administered through the anorectal route. It has a multidimensional effect [10]. Among the different types of *vasti*, *madhutailika vasti* regulates the vitiated *vata dosha* at its site and balances other *doshas*, mainly *kapha dosha* [11] (Table 1). *Rasnasaptakam kwath* is a polyherbal formulation that contains 8 medicinal plants, namely, *Pulchea lanceolata, Tribulus terrestris, Tinospora cordifolia, Boerhavia diffusa, Ricinus communis, Cedrus deodara, Cassia fistula, and Zingiber officinale* (Table 2). It is effective in *grudhrasi* because of its specific effects on the *jangha* (calf), *uru* (thighs), *prishta* (low back), *trika* (sacral region), and *parswa* (flanks) shula. *Vatari guggulu* is an Ayurvedic formulation containing *Terminalia chebula, Terminalia bellerica, Emblica officinalis*, purified sulfur, purified resin of *Commiphora mukul*, and castor oil (Table 3). It is also specifically indicated for sciatica-like conditions [12,13]. *Tila taila* (sesame oil) is a plant-derived oil with anti-inflammatory properties. In Ayurveda, it is considered the best drug to pacify *vata* [14]. The selected Ayurvedic treatment protocol was developed based on classical Ayurvedic principles; contemporary clinical practice patterns; and the pathophysiological understanding of lumbar radiculopathy as a predominantly *vata*-dominant disorder associated with pain, stiffness, inflammation, restricted movement, and functional impairment. The protocol was intentionally designed as a multimodal and whole-system intervention rather than a single-drug approach, reflecting routine Ayurvedic clinical practice. The protocol was therefore selected as a rational, practice-based multimodal regimen intended to address multiple components of lumbar radiculopathy simultaneously.

**Table 1:** Ingredients of madhutailika vasti.

Ingredients	Part used	Botanical name or English name	Family	Quantity (for 3 days)
*Makshika*	—^a^	Honey	—	200 g
*Saindhava*	—	Rock salt	—	12 g
*Taila*	—	Oil	—	200 mL
*Kalka*-*shatahwa*	Seed	*Anethum sowa* Roxb. ex Fleming	*Apiaceae*	24 g
*Kwatha-eranda*	Root	*Ricinus communis* L.	*Euphorbiaceae*	400 mL
*Madanaphala*	Fruit	*Xeromphis spinosa* (Thunb.) Keay	*Rubiaceae*	6 g

^a^Not applicable.

**Table 2:** Ingredients of *rasnasaptakam kwath*.

Ingredients	Sanskrit name	Quantity (part)	Parts used
*Pluchea lanceolata* Oliver & Hiern	*Rasna*	1	Root
*Tinospora cordifolia* (Willd) Hook f & Thomson	*Amrita* (*guduci*)	1	Stem
*Cassia fistula* Linn	*Aragvadha*	1	Fruit pulp
*Cedrus deodara* (Roxb ex D Don) G Don	*Devadaru*	1	Heartwood
*Tribulus terrestris* Linn	*Trikantaka* (*gokshura*)	1	Fruit
*Ricinus communis* Linn	*Eranda*	1	Root
*Boerhavia diffusa* Linn	*Punarnava* (*rakta punarnava*)	1	Root

**Table 3:** Ingredients of the *vatari guggulu* tablet.

Ingredients	Sanskrit name	Quantity (part)	Parts used
*Ricinus communis* Linn	*Vataritaila (eranda*)	1	Seed
Sulfur	*Gandhaka-suddha*	1	—^a^
*Commiphora mukul* (Hook ex Stocks) Engl	*Pura* (*guggulu*)-*suddha*	1	Resin
*Terminalia chebula* Retz	*Haritaki*	1	Pericarp
*Terminalia bellirica* (Gaertn) Roxb	*Bibhitaka*	1	Pericarp
*Phyllanthus emblica* Linn	*Amalaki*	1	Pericarp

^a^Not applicable.

In conventional medicine, lumbar radiculopathy is treated with conservative management such as physiotherapy, including pelvic traction, epidural steroid injections, and surgery. The first line of treatment includes conservative management, including patient education, manual therapy, and nonsteroidal anti-inflammatory drugs (NSAIDs). Most of the patients prefer conservative treatment such as pelvic traction over surgery because it carries a lower risk of complications and lower costs [15,16]. In pelvic traction, the mechanism of action to relieve pain is to separate the vertebrae, thereby reducing pressure or contact forces on injured tissues while increasing peripheral circulation through a massage effect and reducing muscle spasm [17]. In this study, our objectives are to compare the effects of an Ayurvedic treatment protocol with standard care, including an NSAID (diclofenac sodium tablet), Gabaneuron tablet, local application of diclofenac gel, and pelvic traction, on pain and functional disability; to evaluate the change in the quality of life of participants and the need for analgesic medication; and to clinically evaluate the safety of the Ayurvedic treatment protocol compared to standard care in patients with lumbar disc herniation with radiculopathy.

## Methods

### Study Design and Study Setting

The study is an open-label, randomized controlled, parallel group clinical trial conducted at the National Ayurveda Research Institute for Panchakarma (NARIP), Cheruthuruthy, Thrissur district, Kerala, India. The schedule of screening, enrollment, intervention, assessments, and follow-up visits in the clinical trial is shown in Table 4.

**Table 4:** Schedule of screening, enrollment, intervention, assessments, and follow-up visits in the clinical trial.

	Screening	Baseline	Day 8	Day 30	Day 60
Determine eligibility based on the inclusion and exclusion criteria	✓				
Provision of participant information sheet	✓				
Informed consent	✓				
Recording of ODI^a^ score	✓	✓	✓	✓	✓
Demographic and medical history	✓	✓			
MRI^b^ investigation	✓				
Laboratory investigations	✓				✓
General physical examination	✓				
Clinical examination	✓	✓	✓	✓	✓
Assessment of subjective parameters		✓	✓	✓	✓
Drug compliance assessment			✓	✓	
Rescue medication assessment			✓	✓	✓
Adverse event assessment			✓	✓	✓

^a^ODI: Oswestry Disability Index.

^b^MRI: magnetic resonance imaging.

### Study Participants

#### Inclusion Criteria

Participants of either gender aged 25 to 55 years, with an Oswestry Disability Index (ODI) [18] score between 21% and 60% and a diagnosis of lumbar disc herniation with radiculopathy due to intervertebral disc herniation confirmed by magnetic resonance imaging (MRI), who are willing to provide written informed consent prior to participation in the study and adhere to the study protocol, will be included.

#### Exclusion Criteria

The following exclusion criteria will be used for this study: (1) indication for surgical intervention for disc herniation such as severe motor deficit (motor power of lower limbs assessed through the Medical Research Council Manual Muscle Testing scale with a score ≤3), severe spinal stenosis, excruciating pain that cannot be managed by conservative treatment, foraminal stenosis, conjoint nerve root, or perineural cyst; (2) patients who have received nonpharmacological interventions such as physiotherapy, traction, and manual therapy for the management of lumbar disc herniation in the last 3 months; (3) history of or evidence of any of the following: osteoporotic lumbar fracture; presence of inflammatory or infective diseases that affect spinal morphology, such as ankylosing spondylitis, spondylodiscitis or inflammatory spondylitis, or spondylolisthesis; Pott spine; pyriformis syndrome; sacroiliitis; or neural foraminal stenosis; (4) history of spinal surgery in the last 2 years or having epidural fibrosis; (5) patients with cauda equina syndrome, neurological deficits such as foot drop, limb muscle wasting, bowel or bladder incontinence, or nonambulatory patients with monoplegia, paraplegia, or hemiplegia; (6) evidence or history of spinal trauma or spinal malignancy; (7) presence of other medical conditions presenting with numbness and pain in lower extremities, such as diabetic polyneuropathy and peripheral vascular disease, motor neuron disease, multiple sclerosis, stroke, or cognitive impairment; (8) major coexisting medical condition, such as cancer, chronic obstructive pulmonary disease, cardiovascular disease, and severe hepatic and renal dysfunction; (9) peptic ulcer disease and gastrointestinal hemorrhage or perforation; (10) obesity (BMI ≥30 kg/m^2^); (11) metallic implants such as pacemakers and hearing aid implants and other contraindications for MRI; and (12) any other condition that, in the investigator’s opinion, contraindicates the intervention under study.

### Study Intervention

Participants in both groups will be admitted to the inpatient department of NARIP. Participants in the intervention group will receive the Ayurvedic treatment protocol, which includes *madhutailika vasti* for 7 days. As part of the treatment protocol, *abhyanga* (therapeutic massage) using *tila taila* will be performed by the medical team, followed by moist heat. *Madhutailika vasti* will then be administered through the anal route using sterilized equipment. The treatment will be administered from baseline to day 7. The participant will receive education about therapeutic massage with *tila taila* and will be asked to continue until day 30. The oral medications *rasnasaptakam kwath*, along with 1 g of *shunti choorna* (dried powder of *Zingiber officinale* Roscoe) and a 500-mg *vatari guggulu* tablet, will be given twice daily for 30 days from baseline.

The ingredients for *madhutailika vasti* and the sesame oil for therapeutic massage were manufactured at the Good Manufacturing Practices–certified pharmacy at NARIP. The *rasnasaptakam kwath* powder, *shunti choorna* powder, and *vatari guggulu* tablet were manufactured by Indian Medicinal Pharmaceutical Corporation Ltd, Uttarakhand, India, in accordance with the respective standards specified in the Ayurveda Pharmacopoeia of India [19].

Participants in the control group will receive physiotherapy with pelvic traction, adjusted according to the participant’s body weight, for 8 to 9 hours using either a continuous or intermittent method based on the severity of pain. Additionally, a Gabaneuron tablet (300 mg) will be administered twice daily for 30 days from baseline, and diclofenac gel will be applied externally for 30 days. A diclofenac sodium tablet (50 mg-75 mg) will be administered, as advised by a coinvestigator (a practitioner of conventional medicine) or the conventional medicine consultant at NARIP, for a maximum period of 7 days, depending on the severity of pain. Participants will be advised to take the diclofenac sodium tablet only when painful episodes occur during the follow-up period. A pantoprazole tablet (40 mg) will also be advised whenever a diclofenac sodium tablet is administered. The details of the interventions in both groups are shown in Table 5.

**Table 5:** Details of interventions in both groups.

Groups and interventions			Dose, time of administration, or *anupana*		Duration (days)
**Group A**					
	*Rasnasaptakam kwath*	96 mL, 45 min before food, along with shunti choorna (1 g)		30	
	*Vatari guggulu*	500 mg twice a day along with kwath		30	
	*Abhyanga* with lukewarm *tila taila* followed by moist heat	30 mL/d, 30 min daily once		30	
	*Madhutailika vasti*	Makshika 2 prasruta and 1 karsha (approximately 200 g) Sanidhavalavana 1 karsha (12 g) Murchita tila taila 2 prasruta and 1 karsha (approximately 200 mL) Satahwa kalka half pala (24 g) Eranda moola kashaya 4 prasruta and 2 karsha (approximately 400 mL) 1 madanaphala (approximately 6 g)		7	
**Group B**					
	Diclofenac sodium tablet	50-75 mg twice a day after food with lukewarm water		7 (and as needed if painful episodes occur during follow-up)	
	Pantoprazole tablet	40 mg (1-0-0) 30 min before food with lukewarm water		7 (and while taking diclofenac tablets)	
	Gabaneuron tablet	300 mg twice a day after food with lukewarm water		30	
	Local application—diclofenac gel	As necessary		30	
	Physiotherapy—pelvic traction	Continuous or intermittent, as per severity		7	

### Outcome Measures

Changes in the numeric pain rating scale, visual analog scale (VAS) score, and the ODI (version 2.0) score will be assessed as the primary outcome measures. These outcomes will be assessed at baseline (day 1) and on day 8 (after completing IP treatment), day 30, and day 60. The investigators will ask the patients to rate their pain intensity at each visit using a 0 to 10 numeric pain rating scale. The investigators will also record the ODI score using the ODI (version 2.0) during screening. The investigators will instruct the participants on how to fill out each question.

The secondary outcome measure is quality of life, assessed using the 36-item Short Form Health Survey (SF-36) at baseline and on day 8, day 30, and day 60. The occurrence of adverse events (AEs) and the need for rescue analgesic medication, if any, will be recorded on day 8, day 30, and day 60.

### Safety Outcomes

During each follow-up visit, the investigator will seek information on AEs, which will be recorded in the case report form (CRF), and appropriate treatment will be provided. Any serious adverse event (SAE) that occurs after the study period and is considered possibly related to the study treatment will be recorded and reported immediately.

### Withdrawal Criteria

Participants who wish to withdraw from the study for any reason, or who demonstrate any noncompliance, will be withdrawn from the study. Participants will also be withdrawn if a suspected adverse drug reaction, AE, or SAE is confirmed by a causality assessment to be related to the trial intervention. If, during the study, a participant meets any of the exclusion criteria (either newly developed or not previously recognized), the participant will be withdrawn from the study. The reasons for withdrawal will be recorded in the participant’s CRF.

### Sample Size

The sample size for this trial is 80 participants. A previously published study reported a mean change of 5 points in the ODI score from baseline after treatment in the control group [20]. We hypothesized that the intervention group would have a 10-point change in ODI score from baseline after treatment. With an SD of 7 points, 31 participants in each group will be needed to detect the difference with 80% power and 95% CI. Because the sample size estimated using the ODI was larger and more conservative compared to the other coprimary outcome measures, it was considered sufficient to provide adequate statistical power for primary outcomes. Adding an attrition rate of 25%, the sample size becomes 40. Therefore, a total of 80 patients will be enrolled in the trial (40 in each group).

### Recruitment

Participants presenting with features of lumbar radiculopathy at the outpatient department of NARIP will be screened for eligibility to participate in the clinical trial. The written informed consent of the patients will be obtained before screening. Participants with an ODI score of 21% to 60% will undergo MRI of the lumbosacral spine to confirm the diagnosis. A hemogram, liver function test (LFT), renal function test (RFT), and testing for C-reactive protein (CRP), hemoglobin A_1c_, random blood sugar, rheumatoid factor, and human leukocyte antigen (HLA) B27 will be performed at screening. The data will be entered into the CRF for screening, and on day 60, hemogram, LFT, RFT, and CRP will be performed.

### Randomization and Allocation Concealment

The trial is designed as a single-center, open-label, randomized controlled clinical study. Eligible participants will be randomized to the intervention or control group using a randomized block design. Allocation concealment will be achieved using sequentially numbered opaque sealed envelopes, which were prepared by an individual not involved in participant recruitment. The envelopes were identical, opaque, properly sealed, and sequentially numbered to prevent prediction of group allocation. They were stored securely and opened sequentially only after participant enrollment. We have also clarified the procedures followed to maintain the integrity and verification of the concealment process throughout the study.

The details of the study procedure are presented in Figure 1.

**Figure 1:**
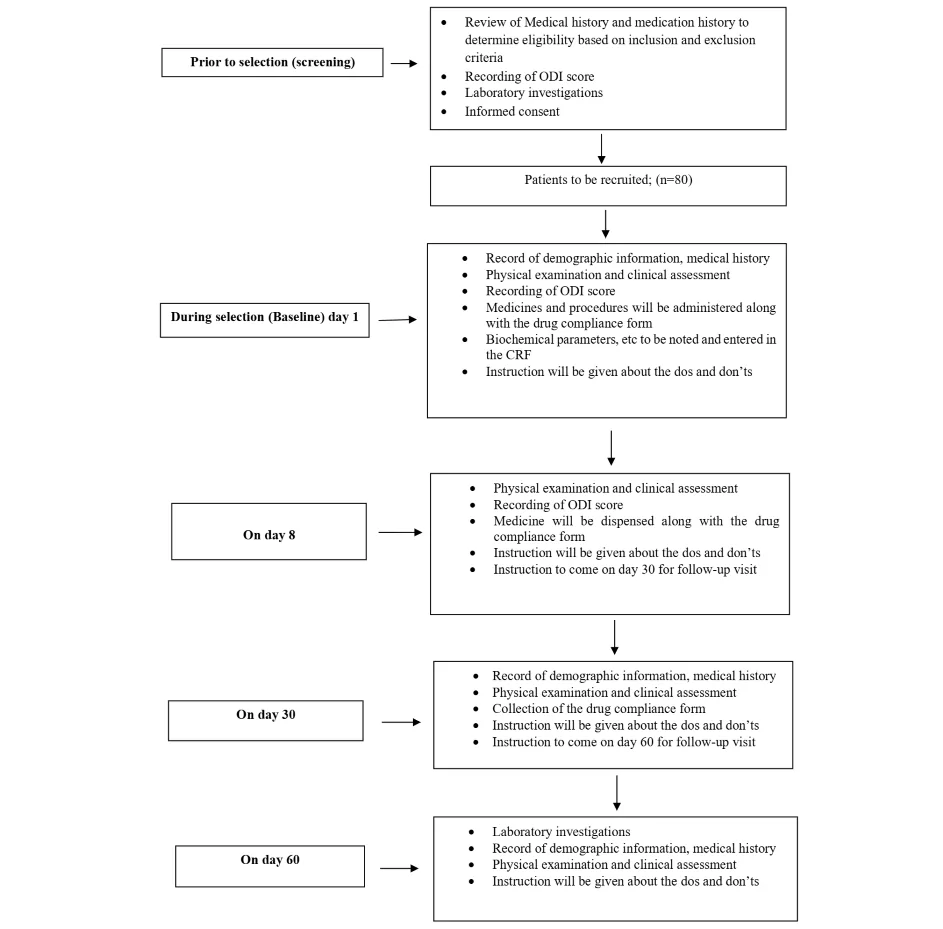
Flowchart of the study. CRF: case report form; ODI: Oswestry Disability Index.

### Compliance

A compliance assessment form will be provided to participants to monitor adherence to the prescribed medicines at each visit. Participants will be instructed to complete the assessment form after each administration of medicine. The completed forms will be collected during follow-up visits.

### Concomitant and Rescue Medication

Participants will be asked to inform the investigators about any concomitant medications they are taking. The investigators will record the details of such medicines and the reason for taking them in the CRF. If there is any medical emergency, rescue medicines can be used, and the details will be recorded in the CRF.

### Data Collection and Documentation

The investigators and the research team will be trained in Good Clinical Practice (GCP) protocols, trial-specific process, and documentation before commencing the study. The research team will collect the information, and the details will be filled in the CRF (Figure 1). All the documented data entered by the team will be checked regularly by the principal investigator (PI) or coinvestigators. Any modifications or corrections made will be made clearly visible, and the corrections will be signed and dated by the PI or coinvestigator. The data will subsequently be entered in an electronic format of CRF and verified as and when required. The original CRF will be archived.

### Statistical Analysis

The collected data will be checked for accuracy and completeness prior to analysis. Categorical variables will be presented as number (percentage) and compared between groups using the chi-square test or Fisher exact test, as appropriate. Continuous variables will be expressed as mean (SD) for normally distributed data or median (IQR) for nonnormally distributed data. Normality will be assessed using probability plots and the Shapiro-Wilk test. The primary analysis of repeated outcome measurements across follow-up visits will be performed using repeated-measures ANOVA for normally distributed continuous variables. In case of violation of normality assumptions or for correlated nonnormal outcomes, generalized estimating equation (GEE) models with appropriate link functions and working correlation structures will be applied. The analysis model will include treatment group, time, and group×time interaction effects to assess differential treatment responses over time.

Between-group comparisons at individual time points will be performed using an independent 2-tailed *t* test or Mann-Whitney *U* test, while within-group changes will be analyzed using a 2-tailed paired *t* test or Wilcoxon signed-rank test, depending on data distribution. Missing data and dropouts will be managed using an intention-to-treat approach, wherever feasible. For repeated-measures analyses using GEE models, all available observations will be used under the assumption of missing at random. The reasons for dropout or missing observations will be documented and reported. Sensitivity analyses may also be performed to assess the impact of missing data on study outcomes. A 2-sided *P* value of <.05 will be considered statistically significant. Statistical analysis will be performed using Stata/MP (version 16.1; StataCorp).

### Monitoring

The sponsor will set up a monitoring committee, which will conduct virtual and on-site monitoring to ensure adherence to the clinical trial protocol and compliance with GCP guidelines and the research policy of the Central Council for Research in Ayurvedic Sciences.

### Trial Audit

The regulatory authorities, the institutional ethics committee (IEC), or the funding agency will audit the trial, and the research team will ensure access to all the documents related to the study for the on-site audit.

### Ethical Considerations

The study was approved by the IEC of NARIP (F. No. 8/16/23/NARIP/Tech meeting/2509; dated March 31, 2023) and has been registered prospectively at the Clinical Trial Registry of India (CTRI/2023/05/052859). The study will be conducted in accordance with the Indian Council for Medical Research National Ethical Guidelines for Biomedical and Health Research on Human participants (2017). Written informed consent (either in English or Malayalam) will be obtained from eligible participants before screening. All protocol modifications will be shared with the IEC and funding agency and will be implemented in the study after the IEC approval. The CRFs will be stored in a secure area and will ensure confidentiality. Routine medical care will be given to study participants, if required, after the completion of study period. The study participants will be compensated with an incidental support of Rs 100 (US $1.05 as of August 25, 2026) for each visit. The study participants are insured under clinical trial insurance.

## Results

The study was funded in March 2023, and the study period is 36 months. Participant enrollment began in November 2023. A total of 136 participants were screened, of whom 80 were enrolled in the study. Data analysis is in progress, and the study outcomes are expected to be published by December 2026.

## Discussion

### Anticipated Findings

Lumbar disc herniation frequently occurs at the L4-L5 or L5-S1 levels, and radicular pain is one of the most common and disabling symptoms [21-24]. The study described here will compare an Ayurvedic treatment protocol to standard care for lumbar disc herniation with radiculopathy, including pelvic traction, oral medication, and topical medication.

Conventional pharmacological management of lumbar disc herniation with radiculopathy includes NSAIDs such as diclofenac and neuropathic agents such as gabapentin. While NSAIDs effectively reduce inflammatory pain, their impact on neuropathic pain is limited [25]. Gabapentin may reduce radicular pain but can cause AEs [26]. Topical diclofenac gel application causes significant reduction in pain; however, its use is associated with local AEs such as skin irritation [27,28].

Although mechanical traction is commonly used and can effectively relieve lumbar and leg pain and improve ODI scores, it has no significant effect on spinal mobility. Its beneficial effects on pain relief are observed when added to medication or bed rest, but its long-term benefits remain inconsistent [29]. Furthermore, systematic reviews indicate that the overall quality of evidence supporting traction is limited, highlighting the need for further comparative evaluation [30]. Ayurvedic management of lumbar radiculopathy includes internal medications, external therapies, and *panchakarma* procedures [31]. Preliminary evidence suggests that the proposed Ayurvedic intervention, namely *rasnasaptakam kwath*, exhibits anti-inflammatory activity [32]. *Vatari* *guggulu* contains *guggulu* as a key ingredient, and pharmacological studies have demonstrated that *guggulu* possesses significant anti-inflammatory and analgesic properties by inhibiting inflammatory mediators and modulating cytokine pathways [33]. Oil massage and therapeutic steaming are well-established Ayurvedic therapies for promoting well-being [34]. As *grudhrasi* is considered a *vatavyadhi*, *vasti karma* is regarded as the most suitable *panchakarma* therapy.

### Strengths

This randomized clinical trial will evaluate the efficacy of Ayurvedic treatment protocol for lumbar disc herniation with radiculopathy in comparison with standard care. The measures used in this clinical trial will yield valid data for the treatment of lumbar disc herniation with radiculopathy.

### Limitations

The study is being conducted at a single center; therefore, participants may be recruited from a single geographical location. The findings are expected to be applicable to countries where Ayurveda is practiced. Hence, the outcomes of this study cannot be generalized. However, regulatory approval for use of the Ayurvedic treatment and availability of trained personnel will be essential. The study is being conducted as an open-label trial because blinding is not feasible for a multimodal Ayurvedic inpatient intervention. As the primary outcomes (VAS, ODI, and SF-36) are patient reported, they may be influenced by treatment expectations, preferences, and attention effects. To reduce bias, outcome assessments will be performed by an independent blinded assessor. Future double-blind randomized trials incorporating objective measures such as MRI findings or biomarkers are recommended to validate these results.

### Conclusions

The outcome of this trial may provide scientific evidence regarding the safety and efficacy of the Ayurvedic treatment protocol in lumbar disc herniation with radiculopathy. The study may also contribute to the development of evidence-based integrative management strategies and standardized treatment protocols for this condition.

## Abbreviations

AE: adverse event
CRF: case report form
CRP: C-reactive protein
GCP: Good Clinical Practice
GEE: generalized estimating equation
HLA: human leukocyte antigen
IEC: institutional ethics committee
IP: inpatient
LFT: liver function test
MRI: magnetic resonance imaging
NARIP: National Ayurveda Research Institute for Panchakarma
NSAID: nonsteroidal anti-inflammatory drug
ODI: Oswestry Disability Index
PI: principal investigator
RFT: renal function test
SAE: serious adverse event
SF-36: 36-item Short Form Health Survey
VAS: visual analog scale

## Data Availability

The data related to the study outcomes will be made available by the funding agency upon reasonable request.
